# Accelerated inbreeding depression suggests synergistic epistasis for deleterious mutations in *Drosophila melanogaster*

**DOI:** 10.1038/s41437-019-0263-6

**Published:** 2019-09-02

**Authors:** Sara Domínguez-García, Carlos García, Humberto Quesada, Armando Caballero

**Affiliations:** 1 Departamento de Bioquímica, Genética e Inmunología, Universidade de Vigo, 36310 Vigo, Spain; 2 Centro de Investigación Marina (CIM-UVIGO), Universidade de Vigo, 36310 Vigo, Spain; 3 CIBUS, Universidade de Santiago de Compostela, 15782 Santiago de Compostela, Galicia Spain

**Keywords:** Quantitative trait, Evolutionary genetics, Quantitative trait, Evolutionary genetics

## Abstract

Epistasis may have important consequences for a number of issues in quantitative genetics and evolutionary biology. In particular, synergistic epistasis for deleterious alleles is relevant to the mutation load paradox and the evolution of sex and recombination. Some studies have shown evidence of synergistic epistasis for spontaneous or induced deleterious mutations appearing in mutation-accumulation experiments. However, many newly arising mutations may not actually be segregating in natural populations because of the erasing action of natural selection. A demonstration of synergistic epistasis for naturally segregating alleles can be achieved by means of inbreeding depression studies, as deleterious recessive allelic effects are exposed in inbred lines. Nevertheless, evidence of epistasis from these studies is scarce and controversial. In this paper, we report the results of two independent inbreeding experiments carried out with two different populations of *Drosophila melanogaster*. The results show a consistent accelerated inbreeding depression for fitness, suggesting synergistic epistasis among deleterious alleles. We also performed computer simulations assuming different possible models of epistasis and mutational parameters for fitness, finding some of them to be compatible with the results observed. Our results suggest that synergistic epistasis for deleterious mutations not only occurs among newly arisen spontaneous or induced mutations, but also among segregating alleles in natural populations.

## Introduction

Epistasis is expected to arise from the interaction of genes in complex biological networks whose expression is tightly regulated and coordinated (Pérez-Pérez et al. 2009; de Visser et al. 2011; Hsuan-Chao Chiu et al. 2012; Sohail et al. 2017), and may have important consequences for a number of issues in quantitative genetics and evolutionary biology (Wagner et al. 1998; Wolf et al. 2000). For example, synergistic (reinforcing, narrowing or negative) epistasis for deleterious alleles, by which the detrimental effects of alleles are enhanced because of interactions between loci, is very relevant for the mutation load paradox and a deterministic mechanism for the evolution of sex and recombination (Charlesworth 1990; Kondrashov 1988, 1994; Barton 1995; West et al. 1998; Roze and Lenormand 2005; de Visser and Elena 2007; Sohail et al. 2017). Synergistic epistasis may also have an impact on the additive genetic variance in bottlenecked populations (Goodnight 1988; López-Fanjul et al. 2002, 2006; Pérez-Figueroa et al. 2009; Ávila et al. 2014) and on the response to artificial selection (Hill 2017). The demonstration of synergistic epistasis for fitness, however, is an elusive task, as natural selection may easily purge or lead to very low frequencies highly detrimental interacting genotypes.

Evidence of synergistic epistasis for deleterious mutations can be obtained from mutation-accumulation experiments, i.e., experiments where an isogenic or highly inbred population, initially devoid of variation, is maintained for a long time so that spontaneous mutations accumulate, or alternatively these are induced by mutagens. Synergistic epistasis for deleterious mutations is expected then to produce an accelerated decline in fitness as mutations accumulate over generations (Crow and Kimura 1970, p. 80). A number of experiments of this type have shown evidence of synergistic epistasis (e.g., Kitagawa 1967; Mukai 1969; Elena and Lenski 1997; Whitlock and Bourguet 2000; Fry 2004; Szafraniec et al. 2003; Rivero et al. 2003; Sanjuan and Elena 2006; Ávila et al. 2006; Dickinson 2008). However, other mutation-accumulation studies have failed to detect synergistic epistasis or have reported variable results (Kibota and Lynch 1996; de Visser et al. 1997; Shabalina et al. 1997; Kondrashov 1998; Elena 1999; Fry et al. 1999; García-Dorado et al. 1999; Keightley and Eyre-Walker 2000; Fry 2004; Kouyos et al. 2007; Halligan and Keightley 2009). In any case, it is possible that the interacting spontaneous or induced mutations found in mutation-accumulation experiments (which are often led to fixation in these experiments) may not be those actually segregating, or do so at very low frequencies, in natural populations. Thus, demonstrating epistasis for naturally segregating alleles should be important for showing the relevance of epistasis in nature.

Evidence of synergistic epistasis for naturally segregating alleles can be obtained from inbreeding depression experiments. Assuming an exponential quadratic model of fitness, it has been theoretically deduced that synergistic epistasis among the fitness effects of deleterious mutations can explain the levels of inbreeding load observed in Drosophila (Charlesworth 1998). In fact, Charlesworth (1998) also showed that a large interaction term at the level of inbreeding effects can arise from a modest quadratic term in the fitness function, which suggests that inbreeding depression experiments should be able to identify synergistic epistasis.

Inbreeding depression may occur either under the partial dominance hypothesis, where deleterious recessive allelic effects are exposed in homozygosis in inbred lines, or under the overdominance hypothesis, which implies heterozygote advantage for fitness. It is unclear what is the proportional contribution of both sources of inbreeding depression. It has been inferred that balancing selection appears to make a large contribution to genetic variation in fitness components in *Drosophila* (Charlesworth 2015), and this may contribute significantly to inbreeding depression. However, genomic data suggests that loci with heterozygote advantage must be considered only a small minority of all loci in a species (Roff 2001; Hedrick 2012). Assuming a model of inbreeding depression caused by the increase in the frequency of homozygous deleterious mutations by inbreeding, a linear decline of fitness with the inbreeding coefficient implies absence of epistasis whereas synergistic epistasis is expected to produce an accelerated decline in fitness (Lynch and Walsh 1998). However, detecting epistasis with inbreeding is not straightforward because of several reasons. First, because the dependence of data on increasing *F* levels, i.e., the fact that the same lines are analysed at the different inbreeding levels. Second, because inbred lines are always lost over time as a consequence of inbreeding depression. And third, because inbreeding increases the elimination of deleterious recessive mutations by exposing them in homozygosis (genetic purging; Wang et al. 1999; Hedrick and García-Dorado 2016). The three reasons result in bias against detecting non-linearity. The first, because an obvious correlation is expected between the means of the lines in consecutive generations. The second, because extinct lines are expected to show low fitness values in generations previous to extinction (Roff 2001; Lynch and Walsh 1998). And the third, because synergistic epistatic mutations are more likely to be removed, implying a deceleration in the rate of decline in fitness, which erases the footprint of epistasis.

The majority of analyses with highly inbred lines (full-sib lines) generally suggest a linear relationship between fitness and the inbreeding coefficient (see Willis 1993; Falconer and Mackay 1996, p. 40; Roff 2001, p. 320; Lynch and Walsh 1998, p. 255), although some occasional evidence of synergistic epistasis from inbreeding experiments has been observed, for example, in *Mimulus guttatus* (Willis 1993), gymnosperms (reviewed by Charlesworth and Charlesworth 1987), and *D. melanogaster* (Rosa et al. 2005). Pekkala et al. (2014) observed a decelerated decline in egg-to-adult viability for full-sib lines of *Drosophila littoralis*, implying the absence of synergistic epistasis, but found an accelerated decline in the last (sixth) generation of full-sib mating for offspring fecundity, which could be compatible with synergistic epistasis. Likewise, Salathé and Ebert (2003) found no change in fitness in *Daphnia magna* with intermediate values of inbreeding (*F* ≥ 0.25 and 0.5) but a sharp decline with the highest *F* ( ≥ 0.75), which would suggest synergistic epistasis, although there has been some debate about its interpretation (Trouve et al. 2004; Ebert et al. 2004). Kelly (2005), using crosses between highly inbred lines of *Mimulus guttatus*, also found a non-linear decline of pollen viability with inbreeding coefficient and some evidence of epistasis (but not of synergism) for other morphological and developmental traits. More recently, Sharp and Agrawal (2016) performed an experiment combining the accumulation of mutations induced by ethyl methane-sulphonate in *D. melanogaster* and the establishment of lines at different inbreeding levels, showing evidence of synergistic epistasis between induced mutations.

Here, we show results from two laboratory experiments using full-sib lines founded from two different natural populations of *Drosophila melanogaster*, which show a consistent accelerated decline for fitness, pointing towards synergistic epistasis among naturally segregating deleterious alleles. We also propose a model of epistasis able to explain the empirical results.

## Materials and methods

### Experimental procedure

#### Base populations

Two experiments (I and II) were carried out using different *D. melanogaster* populations captured in different years at two localities of Galicia (northwestern Spain) separated by about 20 km. For Experiment I, ∼1000 individuals were collected in 2006 in a wine cellar at Beade (Vigo), and maintained under laboratory conditions during 103 generations in ~30 bottles (30–60 flies per bottle) with circular mixing of the bottles until the start of the experiment in October 2011. Experiment II base population was founded from a sample of ∼1000 individuals taken in October 2013 from another wine cellar (at Salvaterra do Miño) and maintained in the laboratory during 12 generations, again with circular mixing of ~50 bottles (40 males and 40 females per bottle), until the start of the experiment in June 2014. Thus, one population was relatively close to its natural constitution (Exp II), whereas the other was a long-term established laboratory population (Exp I). Flies from both base populations and the subsequent experimental lines were reared under standard conditions (baker’s yeast-agar-sucrose medium, continuous light, 25 ± 1 °C temperature, 65 ± 5% relative humidity) and handled at room temperature under CO_2_ anaesthesia. Virgin males and females were used for mating across the entire experiments.

#### Experimental lines

**Fig. 1:**
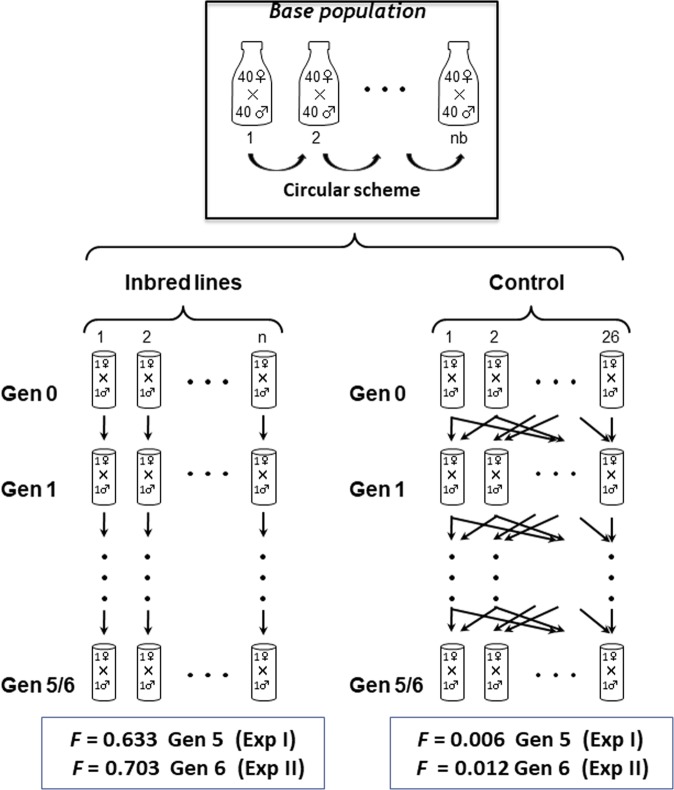
Experimental design and mating scheme to produce and maintain full-sib inbred lines and an outbred control during five (Experiment I) or six (Experiment II) generations, and their corresponding inbreeding coefficient (*F*) reached. *n*: total number of inbred lines (50 for Exp I and 68 for Exp II. nb: Number of bottles of Base Population (30 for Exp I and 50 for Exp II). The control line is maintained with maximum avoidance of inbreeding

To produce the experimental lines, virgin males and females were randomly taken from each base population, placed as breeding couples into separate vials and maintained during two generations with avoidance of consanguineous matings (using flies from different vials) before the start of the experiments. A total of 76 and 94 vials (couples) were finally available for Exp I and II, respectively. From each of them 26 couples were randomly assigned to be an outbred control line, and the remainder to be inbred lines (50 for Exp I and 68 for Exp II). Both control and inbred lines were maintained in vials during 5 (Exp I) or 6 (Exp II) generations following a maximum avoidance of inbreeding (Wright 1921) scheme in the case of the control line, and full-sib (single brother–sister) mating, in the case of the inbred lines (Fig. 1). The expected inbreeding coefficient of the inbred lines in the last generation was 0.633 (Exp I; gen. 5) and 0.703 (Exp II; gen. 6) (see below). The expected inbreeding coefficient in the control line was obtained simulating the genealogy followed by the maximum avoidance of inbreeding design and was 0.006 at generation 5 (Exp I) and 0.012 at generation 6 (Exp II), i.e., very close to zero.

#### Evaluation of fitness

Total productivity of pupae was the trait chosen to assess fitness in both experiments. This is a composite trait including the mating success of the couple, fecundity of the mother and offspring egg-to-pupae viability. The number of pupae present after an 11-day incubation period (thus implying most life-time pupae production) was counted in two vials. Couples were set-up for mating in individual vials and then were passed to new fresh vials after 4 days to avoid too high density in the progeny. Thus, the productivity of the first vial included eggs produced over days 0–3 post-mating and the second vial eggs produced over days 4–11. The total productivity of each couple was the sum of the two vials. In Exp II, a zero value was assigned when the mother was alive after 11 days but there were no pupae in the vial. Mother absence and infertility were not distinguished in Exp I.

The productivity was evaluated at generations 0, 1, 3 and 5 in Exp I and at all generations (0–6) in Exp II. In the last generation, the number of surviving inbred lines was 22 (44%) in Exp I (generation 5) and 23 (34%) (or 17, i.e., 25%, disregarding lines with zero productivity) in Exp II (generation 6).

#### Estimation of inbreeding depression

Inbreeding depression was evaluated by the regression of *W*_I_/*W*_o_ (or its logarithm) on the expected inbreeding coefficient at generation *t*, *F*_*t*_, where *W*_I_ and *W*_o_ are the mean pupae productivity for inbred lines and outbred control, respectively. Ávila et al. (2013) showed that total inbreeding depression rate for pupae productivity (the same trait as evaluated in the current experiments) was 1.19% per 1% increase in inbreeding coefficient. As the trait was analysed using non-inbred mothers and inbred offspring and viceversa, the maternal contribution to inbreeding depression could be quantified, being 0.56%, i.e., about a half of the total inbreeding depression. Thus, in order to estimate inbreeding depression in the experiments it was assumed that about a half of the trait is due to the mother fecundity component and the other half to the progeny viability component. Thus, for the calculation of the inbreeding depression rate, the expected inbreeding coefficient at generation *t* (*F*_*t*_) was calculated as the average of the inbreeding coefficients of mothers and progeny, i.e., *F*_*t*_ = 0, 0.125, 0.313, 0.438, 0.547, 0.633 and 0.703, for generations 0–6, respectively.

To ensure that the estimate of inbreeding depression is not biased because of the differential extinction of the lines, only the surviving inbred lines at the last generation were used in the analysis, as recommended by Lynch and Walsh (1998, pp. 267–268). Akaike and Bayesian Information Criterion (AIC, BIC), and Likelihood Ratio Test (LRT) were performed to assess whether linear or quadratic models fit best to the decline in relative fitness across inbreeding coefficients, using R v. 3.4.2 (R Core Team 2017). Normality of the data was tested with the function *shapiro.test*. The linear and quadratic models were set-up with the functions *lm*(ID ~ *F*) and *lm*(ID ~ *F* + *F*2), respectively, where ID is the inbreeding depression value (ratio *W*_I_/*W*_o_ in raw scale and ln[*W*_I_/*W*_o_] in log scale) and *F* and *F*2 the inbreeding coefficients and their squared values. AIC, BIC and lrtest R functions were used to analyse the fit of the data to the two models.

In addition, to further test the non-linearity of the inbreeding depression, the procedure proposed by Lynch and Walsh (1998, p. 267) was followed both with the raw productivity data and for log scale data. This test intends to detect epistatic effects involving dominance and consists in comparing the change in mean fitness in the lines per increment in coefficient of inbreeding (*F*) between two low levels of *F* and two high levels of *F*, which do not overlap. For example, it may be compared the decline in fitness between *F* = 0 and 0.125 vs. that between *F* = 0.5 and 0.70 and test whether the latter is significantly larger than the former. For Experiment I and the combination of Experiments I and II, the range used was for generations 0–1 vs. 3–5, and for Experiment II for generations 0–2 vs. 4–6. For example, for Exp I, letting *z*_0_, *z*_1_, *z*_3_ and *z*_5_ be the four observed mean values of ln(*W*_I_/*W*_o_) with expected inbreeding coefficients *F*_0_, *F*_1_, *F*_3_ and *F*_5_, the measure of non-linearity is $\Delta I = \frac{{z_1 - z_0}}{{F_1 - F_0}} - \frac{{z_5 - z_3}}{{F_5 - F_3}}$. A conservative estimate of the sampling variance of Δ*I* is ${\mathrm{Var}}\left( {\Delta I} \right) = \frac{{\left[ {{\mathrm{SE}}\left( {z_1} \right)} \right]^2 + \left[ {{\mathrm{SE}}\left( {z_0} \right)} \right]^2}}{{\left( {F_1 - F_0} \right)^2}} + \frac{{\left[ {{\mathrm{SE}}\left( {z_5} \right)} \right]^2 + \left[ {{\mathrm{SE}}\left( {z_3} \right)} \right]^2}}{{\left( {F_5 - F_3} \right)^2}}$, and a test for non-linearity is then $t = \frac{{\left| {\Delta I} \right|}}{{\sqrt {{\mathrm{Var}}\left( {\Delta I} \right)} }}$, which under the null hypothesis of linearity would be *t*-distributed with degrees of freedom equal to the number of lines analysed.

#### Purged inbreeding coefficient

Inbreeding is expected to expose recessive deleterious mutations in homozygosis, accelerating its elimination by selection (genetic purging). As a consequence, the actual inbreeding coefficient may be lower than the expected genealogical one (*F*), and the observed heterozygosity of markers larger than the expected one (Rumball et al. 1994; Demontis et al. 2009; Bersabé et al. 2016). A purged inbreeding coefficient at any generation *t* (*g*_*t*_) can be predicted from the recursive equation of García-Dorado (2012), accommodated for the case of a full-sib line,

$$g_t = \frac{1}{4}\left( {1 + g_{t - 2} + 2g_{t - 1}} \right)\left( {1 - 2dF_{t - 1}} \right)$$

where *F*_*t*_ is the expected genealogical inbreeding coefficient that is obtained recursively for a full-sib line as $F_t = \frac{1}{4}\left( {1 + F_{t - 2} + 2F_{t - 1}} \right)$, and *d* is the purging coefficient, which can be estimated from empirical data. López-Cortegano et al. (2016) estimated the purging coefficient for pupae productivity in a laboratory population of *Drosophila melanogaster* under non-competitive conditions finding a value of *d* ≈ 0.3 for overall inbreeding depression and 0.2 for non-lethal alleles. As most purging in full-sib lines would occur for lethal genes (Wang et al. 1999), we assumed a conservative value of *d* = 0.1, which results in expected purged inbreeding coefficients (again averaging inbreeding values of consecutive generations) of *g*_*t*_ = 0, 0.125, 0.303, 0.405, 0.482, 0.527 and 0.554, for generations 0–6, respectively. These values show the expected restriction in inbreeding because of purging, and inbreeding depression analyses were also made assuming these coefficients.

### Computer simulations

Computer simulations were carried out assuming the same design as the experiments with the objective of investigating the outcomes of different mutation models, including dominance and epistasis. The procedures used in the simulations are explained next.

#### Base population and mutational parameters

Individual-based simulations were carried out to obtain a base population under mutation–selection–drift balance, by allowing for the occurrence of mutations for biallelic loci in a random mating population of size *N*_*b*_ = 1000 monoecious diploid individuals maintained for 10,000 generations. A model of deleterious non-recurrent mutations appearing with rate *U* per haploid genome and generation was assumed. The genotypic fitness values for a given locus *l* (*w*_*l*_) were 1, 1–*h*_*l*_*s*_*l*_ and 1–*s*_*l*_, for the wild-type homozygote, the heterozygote and the mutant homozygote, respectively. Homozygous allelic effects (*s*_*l*_) on fitness were obtained from a gamma distribution with shape parameter *β* and mean value *s*. The dominance coefficient (*h*_*l*_) was assumed to have a negative correlation with the selection coefficient by using the model of Caballero and Keightley (1994), where *h*_*l*_ values are taken from a uniform distribution ranging between 0 and exp(–*ks*_*l*_), *k* being a constant to obtain the desired average value (*h*). Additionally, lethal mutations (*s* = 1) were also considered to appear with rate *U*_*L*_ = 0.015 per haploid genome and generation (Simmons and Crow 1977). Individuals were chosen as parents every generation with a probability according to their fitness. Polygamous mating was assumed. For each mating, a random gamete was chosen from each of the parental individuals assuming free recombination between loci to generate an offspring. This was repeated until all *N*_*b*_ individuals of the population were obtained.

For the non-epistatic (multiplicative) model, the fitness of each individual (*W*_*i*_) was obtained as the product of genotypic fitnesses across all loci *W*_*i*_ = ∏ *w*_*l*_. For the epistatic model, synergistic epistasis was assumed only between mutant homozygotes. As inbreeding increases the frequency of homozygotes at the expense of heterozygotes, assuming epistasis between homozygotes is expected to generate an accelerated fitness decline with inbreeding. The model is one of the simplest ones, and implies the generation of additive-by-additive, additive-by-dominance and dominance-by-dominance epistatic variance (López-Fanjul et al. 2002). Under this premise, two epistatic models were assumed (Table 1):

- *Quadratic homozygous fitness model*, for which the homozygous fitnesses of epistatic loci are squared. That is, if an individual carries *n* ≥ 2 mutant homozygotes the fitness of the individual would be *W*_*ie*_ = *W*_*i* _× ∏ (*w*_*l*_), where the product refers to all homozygous mutations carried by the individual. That is, the fitness of an individual is obtained as the product of the fitness of heterozygous mutations and the squared fitness (if *n* ≥ 2) of the homozygous mutations.
- *High-order homozygous fitness model*, for which the homozygous fitnesses of epistatic loci are raised to a power equal to the total number of homozygous mutations carried by the individual. Thus, if an individual carries *n* ≥ 2 mutant homozygotes the fitness of the individual would be *W*_*ie*_ = *W*_*i* _ × ∏ (*w*_*l*_)^*n*–1^, where the product refers to all homozygous mutations carried by the individual.

**Table 1:** Simulation epistatic models and mutational parameters considered

Model	*U*	*s*	*h*	*β*	*U*_L_	*α*	*ϕ*
Quadratic homozygous fitness	0.1	0.05, 0.1, 0.2	0.2, 0.3, 0.4	2.0	0.015	–	–
	0.5	0.0001, 0.001, 0.01	0.2, 0.3, 0.4	0.2	0.015	–	–
High-order homozygous fitness	0.05	0.05, 0.1, 0.2	0.2, 0.3, 0.4	2.0	0.015	–	–
	0.5	0.0001, 0.001, 0.01	0.2, 0.3, 0.4	0.2	0.015	–	–
Quadratic model based on number of homozygotes and heterozygotes	0.05	–	0.2	–	–	0.005, 0.01, 0.02	0, 0.01, 0.1
	0.5	–	0.2	–	–	0.0001, 0.0005, 0.001	0, 0.001, 0.005

*U* haploid deleterious mutation rate per generation, *s* average homozygous effect of mutations, *h* average dominance coefficient, *β* shape parameter of the distribution of homozygous effects, *U*_L_ lethal mutation rate, *α* parameter for linear fitness decline as a function of the number of homozygous and heterozygous genotypes, *ϕ* parameter for quadratic fitness decline as a function of the number of homozygous and heterozygous genotypes

For both epistatic models, two contrasting mutational parameter values were assumed. One involving a low number of mutations with relatively large effect, and another with many more mutations of smaller effect (Table 1). For the first model, the mutation rate assumed was either *U* = 0.05 or 0.1. These rates are close to the median estimate obtained from mutation-accumulation experiments across eukaryotes (Halligan and Keightley 2009; García-Dorado et al. 2004; Caballero 2017). The assumed mean homozygous effect values were *s* = 0.05, 0.1 and 0.2, with actual values obtained from a gamma distribution with shape parameter *β* = 2, approximately equal to the average estimate gathered by Halligan and Keightley (2009) for eukaryotes excluding estimates not distinguishable from zero or infinity.

For the second model, a much larger deleterious mutation rate was assumed (*U* = 0.5), consistent with the estimate obtained from a comparison between the nucleotide divergences of evolutionary close species (*D. melanogaster* and *D. simulans*) by Haag-Liautard et al. (2007) (see also Charlesworth 2015). The large difference between the former estimates obtained from mutation-accumulation experiments and this molecular-based estimate occurs because mutation-accumulation experiments can only capture mutations of large effect, not having power to detect homozygous effects lower than about 5 × 10^–4^ (García-Dorado et al. 2004). Thus, for this second model, mean effects were assumed to be down to three orders of magnitude lower (*s* = 0.0001, 0.001 and 0.01), and a highly leptokurtic shape of the distribution of effects was considered, with a *β*-value one order of magnitude lower than in the previous model (*β* = 0.2). The dominance coefficient (*h*_*l*_) for both models was assumed as explained above assuming three different mean dominance coefficients (*h* = 0.2, 0.3 and 0.4). For both models, lethal mutations were also considered as described above.

An additional quadratic model of epistasis proposed by Charlesworth (1990) and extended by Charlesworth et al. (1991) was also considered, which is just based on the number of homozygous and heterozygous genotypes. For this model, the fitness of an individual (*i*) is obtained as *W*_*i*_ = exp[−*α*(*n*_1_ + *hn*_2_) – ½*ϕ*(*n*_1_ + *hn*_2_)^2^], where *n*_1_ and *n*_2_ are the number of heterozygous and homozygous deleterious mutations in the individual, respectively, *h* is the coefficient of dominance assumed, and *α* and *ϕ* are positive coefficients that determine the rate of decline in fitness as a function of the number of homozygous and heterozygous genotypes (linear model) or its square (quadratic model), respectively. For this model, the deleterious mutation rates assumed were, again, *U* = 0.05 and 0.5, and *h* = 0.2. The values of *α* chosen in order to obtain a decline in fitness analogous to the observed empirically in the lines were those shown in Table 1. Note that *ϕ* = 0 implies no epistasis.

#### Inbreeding experiment

Samples of two individuals were collected from the base population and subjected to six generations of full-sib mating. The same genetic models (non-epistatic or epistatic) and mutational parameters were applied as for the base populations. To simulate more realistically the productivity of flies, mutations were considered to either affect fecundity or viability (half of each) and a maximum number of progeny per couple was assumed. This value was obtained, for each mating pair, as a deviation from a normal distribution with mean 84 and standard deviation 22, obtained from the observed productivities of the control population vials across generations for the two experiments. Then, the fitness of each individual (*i*) was composed of a fecundity (*f*) and a viability (*v*) component, *W*_*i*_ = *W*_*f,i* _× *W*_*v,i*_, following models considered by Theodorou and Couvet (2015) and Caballero et al. (2017). At a given generation, the number of progeny per couple was obtained as a normal deviate *N*(84,22) × √*W*_*f,x*_ × √*W*_*f,y*_, where *x* and *y* are the parents. The resulting offspring (*i*) survived with probability *W*_*v,i*_. From the total surviving progeny two randomly chosen individuals became the parents for the next generation. The line could become extinct if only one parent or none was available for mating. This process was replicated 1000 times and results were averaged over replicates.

The fecundity and viability of individuals were averaged across replicates each generation. Average fitness (*W*_I_) for the inbred lines at a given generation *t* was obtained as the product of the average fecundity of generation *t*–1 (parents) and the average viability of generation *t* (offspring). Inbreeding depression was then evaluated as the change in –ln(*W*_I_) across *F*_*t*_ values, where, as in the experiments, the expected inbreeding coefficients (*F*_*t*_) are the averages between consecutive generations.

## Results

### Experimental results

**Fig. 2:**
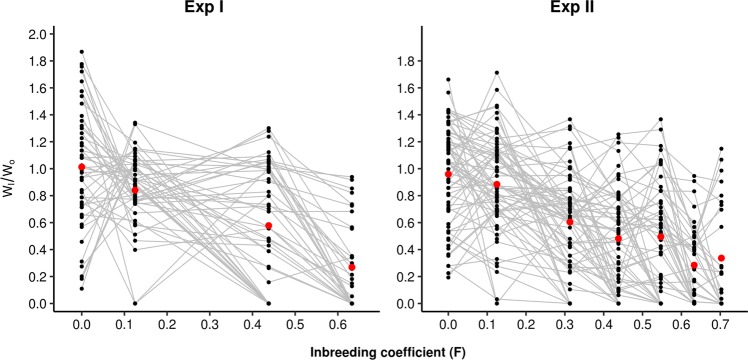
Productivity (*W*_I_) of all inbred lines over generations (expressed as a ratio of the mean productivity of the control line in the corresponding generation, *W*_O_) for Experiments I and II. Note that Exp I productivity was only evaluated in generations 0, 1, 3 and 5. The mean productivities in the control lines were *W*_O_ = 54.61, 87.96, 95.32 and 93.73 for generations 0, 1, 3 and 5 of Exp I, respectively; and *W*_O_ = 93.27, 94.00, 95.85, 81.27, 65.85, 79.29 and 86.21 for generations 0–6 of Exp II, respectively

The productivity of all inbred lines, expressed as a ratio of the mean productivity of the control line, is shown in Fig. 2 across generations for both experiments. The figure shows the observed decline in mean relative fitness by inbreeding depression and the loss of lines over generations. The variance of relative fitnesses was similar in Exp I and Exp II (averages of 0.10 and 0.11 across generations, respectively, without considering the zero values in Fig. 2). There was not any apparent increase in variance between lines over generations: 0.20, 0.04, 0.10 and 0.08 for generations 0, 1, 3 and 5, respectively, for Exp I; and 0.13, 0.11, 0.09, 0.10, 0.12, 0.07 and 0.14 for generations 0–6, respectively, for Exp II.

**Fig. 3:**
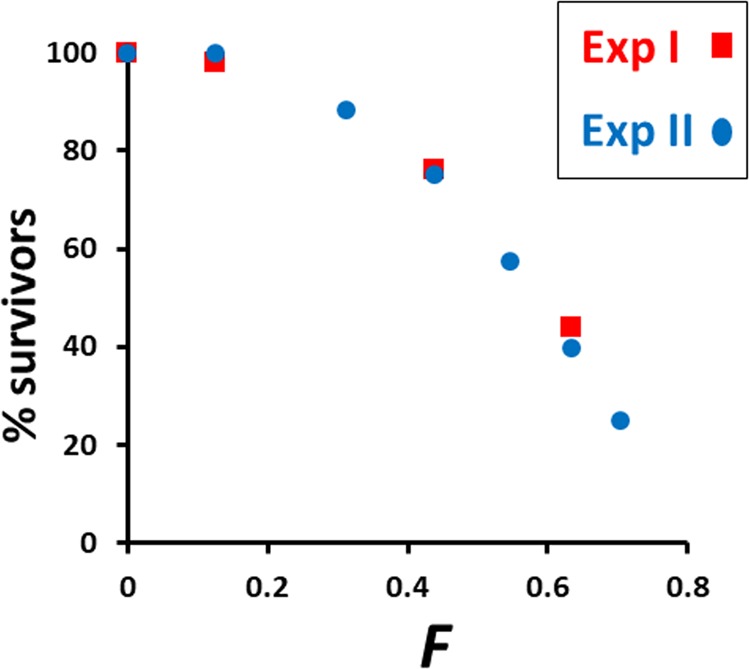
Proportion of surviving full-sib lines across generations as a function of the expected inbreeding coefficient (*F*) for Experiments I (red squares) and II (blue circles). Lines with zero productivity in Exp II are not considered in the calculated proportion

The proportion of full-sib lines surviving across generations was remarkably similar for both experiments (Fig. 3), showing an accelerated rate of extinction with the increase in inbreeding coefficient. We finished the experiments at the fifth and sixth generation for Exp I and II, respectively, as we needed a minimum number of lines to make the analysis, and only 22 and 23 lines, respectively, were left at this point. The estimated purged inbreeding coefficient (*g*) was restricted over generations, as expected, in relation to the expected genealogical *F* (Supplementary Material Fig. S1). In the sixth generation, the expected *F* assumed was 0.703 (average of *F* in generations 5 and 6), whereas the corresponding expected *g* was 0.554.

**Fig. 4:**
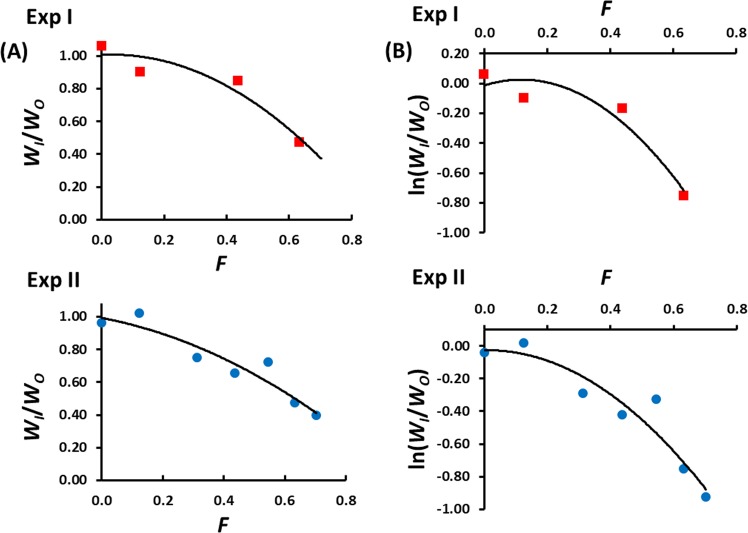
Inbreeding depression for pupae productivity for Experiments I and II, estimated as the ratio between the average productivity of inbred lines (*W*_I_) and the outbred control (*W*_O_) in each generation (**a**) or the logarithm of the ratio (**b**), against the expected inbreeding coefficient (*F*). The data fitted better to a quadratic line (shown) than to a linear one

Figure 4 presents the fitness decline over generations (raw scale in panel a and log scale in panel b) corresponding to the surviving lines at the end of each experiment, against the expected inbreeding coefficient. Supplementary Material Fig. S2 shows the corresponding results when a purged inbreeding coefficient (*g*), rather than the expected genealogical one (*F*), is assumed. Under a multiplicative model of fitness, the mean fitness decline is expected to be decelerated in raw scale, whereas it is expected to be linear in log scale. However, the decline of fitness in both experiments was curvilinear (accelerated) both in raw scale and log scale, i.e., with an increasingly higher decline for large values of the inbreeding coefficient. The linear terms of the quadratic regression on *F* values were all positive, except for the raw data of Exp II, whereas the quadratic terms were all negative (Table S1). The regressions cut the *y*-axis (generation 0) in points very close to 1 (raw scale) and zero (log scale), as expected (Table S1).

The data did not depart from normality except in the case of the two combined experiments in raw scale (Table S2). A comparison of the fit of the fitness decline to linear and quadratic regression models is shown in Table 2. The AIC analyses showed a better fit (lower values of AIC) for a quadratic model of log fitness decline for both experiments and in combination, as well as when *F* or *g* inbreeding coefficients were assumed. Similar results were obtained with BIC and are not shown. The Likelihood Ratio Test showed a significant better fit of the quadratic regression model in log scale for the combined data of both experiments for both *F* and *g* inbreeding coefficients, and also for Exp II for *g* inbreeding coefficients.

**Table 2:** Results from AIC analyses, log likelihoods, and Likelihood Ratio Test Chi-squared and probability values in order to compare the quadratic and linear regression models using inbreeding depression results of Experiments I and II and both combined (I + II)

	Genealogical inbreeding (*F*)			Purged inbreeding (*g*)		
Experiment	I	II	I + II	I	II	I + II
Raw scale						
AIC (linear)	–2.39	–10.52	–18.39	–1.27	–8.29	–15.07
AIC (quad.)	–2.50	–9.90	–19.23	–0.82	–9.00	–17.07
Log Lik. (linear)	4.20	8.26	12.19	3.64	7.14	10.53
Log Lik. (quad.)	5.25	8.95	13.61	4.41	8.50	12.53
LRT Chi-square	2.102	1.380	2.836	1.539	2.709	4.001
LRT *p*-values	0.147	0.240	0.092	0.215	0.100	**0.045**
Log scale						
AIC (linear)	1.52	–2.75	–6.87	2.56	–0.23	–3.31
AIC (quad.)	–0.06	–4.42	–11.45	2.08	–2.19	–7.58
Log Lik. (linear)	2.24	4.37	6.43	1.71	3.11	4.66
Log Lik. (quad.)	4.03	6.21	9.72	2.96	5.09	7.79
LRT Chi-square	3.580	3.675	6.573	2.486	3.964	6.269
LRT *p*-values	0.058	0.055	**0.010**	0.115	**0.046**	**0.012**

Results with data in raw scale or log scale and assuming the genealogical inbreeding coefficients (*F*; Figs. 4A, B) or purged inbreeding coefficients (*g*; Supplementary Figs. S2A, B). Significant LRT *p*-values shown in bold

In addition, the *t*-test of Lynch and Walsh (1998) for non-linearity of fitness decline showed significance (*p*-value < 0.05) or probabilities close to the significance threshold for all tests carried out (Table 3).

**Table 3:** Test for epistasis of Lynch and Walsh (1998) for Experiments I (generations 0–1 vs. 3–5) and II (generations 0–2 vs. 4–6), and both experiments in combination (I + II) (generations 0–1 vs. 3–5)

	Genealogical inbreeding (*F*)			Purged inbreeding (*g*)		
Experiment	I	II	I + II	I	II	I + II
Raw scale						
* ΔI*	0.68	1.40	1.07	1.84	3.81	1.92
* t-*value	1.475	1.822	1.640	2.487	2.470	2.527
Probability	0.077	**0.041**	0.054	**0.010**	**0.011**	**0.007**
Log scale						
* ΔI*	3.08	2.55	2.83	5.37	6.35	4.43
* t-*value	3.004	1.362	2.786	3.261	1.607	3.294
Probability	**0.003**	0.093	**0.004**	**0.002**	0.061	**0.001**

The test is made with data in raw scale or log scale, and assuming the genealogical (*F*) or purged (*g*) inbreeding coefficient. Significant *p*-values shown in bold

#### Simulation results

**Fig. 5:**
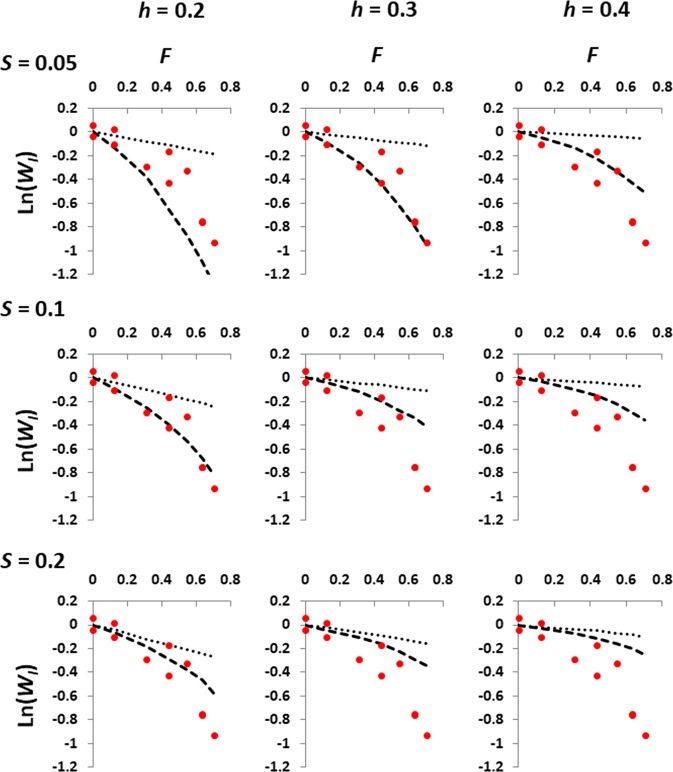
Comparison between simulation results (lines) and experimental data (dots), including Experiments I and II. Each panel shows the decline in log relative fitness, ln(*W*_I_), for increasing values of the expected genealogical inbreeding coefficient (*F*) in the full-sib lines. Averaged simulation results assuming a multiplicative (non-epistatic) model are shown as dotted thin lines, whereas averaged results assuming an epistatic model of variation are shown as broken thick lines. The epistatic model (high-order homozygous fitness model) assumes that homozygous fitnesses of epistatic loci are raised to a power equal to the total number of homozygous mutations carried by the individual if there are more than one. Deleterious mutations are assumed to appear with haploid rate *U* = 0.05, variable effects obtained from a gamma distribution with shape parameter *β* = 2, mean homozygous effect *s* and variable dominance coefficients with mean *h*

The quadratic homozygous fitness model, either assuming a low (*U* = 0.1) or high (*U* = 0.5) mutation rate, produced almost linear or slightly non-linear declines in fitness (Supplementary Figs. S3 and S4), incompatible with the non-linear decline observed in the experimental results (Fig. 4). The high-order homozygous fitness model rendered complete extinction of the lines in the initial generation under the high mutation rate scenario (*U* = 0.5). However, this epistatic model, under the low mutation rate scenario (*U* = 0.05), produced a non-linear fitness decline compatible with that found experimentally (Fig. 5). The epistatic model assumed in these simulations implies that homozygous fitnesses of epistatic loci are raised to a power equal to the total number of homozygous mutations carried by the individual. This number was, on average, between 1.3 and 6.3 in the last generation, depending on the simulated scenarios. The models that apparently fitted best to the empirical data were those with average parameters *s* = 0.05 and *h* = 0.3 and *s* = 0.1 and *h* = 0.2 (Fig. 5). For the last model, the average number of homozygous deleterious genotypes per individual in the full-sib lines was 0.2, 1.5, 2.0, 2.6, 3.0 and 3.4 for generations 1 to 6, respectively. Thus, epistasis was assumed to occur for up to a maximum average of 3.4 loci and the homozygous fitness of these mutations would be elevated to that power. The observed frequency of deleterious homozygous genotypes per individual relative to its value without purging is shown in Supplementary Fig. S5. It is seen that purging reduced the frequency of deleterious homozygotes as expected. Finally, the quadratic model for which fitness decline is a function of the number of heterozygotes and homozygotes also produced linear declines in fitness and was, thus, inconsistent with the experimental results (Supplementary Figs. S6 and S7).

## Discussion

We have shown evidence of a nonlinear decline in fitness upon inbreeding in two populations of *Drosophila melanogaster*, captured in different localities within a time period of about 7 years. The population of Exp I had been maintained in the laboratory for about 100 generations before the inbreeding experiment, whereas that of Exp II was evaluated only 13 generations after its capture, thus being likely to keep its genetic composition close to that of the original natural population. The results of the two experiments, nevertheless, are consistent in suggesting an outcome compatible with a model of synergistic epistasis among deleterious alleles.

A number of mutation-accumulation experiments have shown some evidence of synergistic epistasis (e.g., Mukai 1969; Whitlock and Bourguet 2000; Dickinson 2008). However, epistasis among newly appeared mutations does not necessarily imply that this is frequent in natural populations, because natural selection may eliminate or reduce the frequency of many of the epistatic mutations. In fact, many experiments of inbreeding depression with natural populations show a linear decline in fitness compatible with a multiplicative or additive model of variation (Willis 1993; Roff 2001; Lynch and Walsh 1998), or show only occasional synergistic epistasis (Rosa et al. 2005). The reason for these negative results may be that the detection of a nonlinear decline upon inbreeding can be masked by the non-independence of data and particularly by the loss of individuals and lines over time by selection (Roff 2001; Lynch and Walsh 1998).

There has been, however, a number of inbreeding experiments clearly pointing towards synergistic epistasis. Kelly (2005) found evidence of synergistic epistasis for a pollen size index (the proportion of grains in the larger size category, highly correlated with pollen viability) in *Mimulus guttatus*. In this experiment, lines maintained for seven to nine generations of self-fertilisation (thus with an expected inbreeding coefficient close to one) were crossed in pairs to get F_1_, F_2_ and backcrosses to produce line-cross derivatives with expected inbreeding coefficients of 0, 0.5, 0.75 and 1. As the lines used for the analyses were highly inbred it is expected that they were devoid of lethal or highly deleterious alleles, which had been purged in the inbreeding process (Kelly 2005). More recently, Sharp and Agrawal (2016) followed an approach combining the accumulation of induced mutations and the generation of inbred lines. They generated an isogenic population of *Drosophila melanogaster* mutagenized by EMS and made crosses involving visible markers and balancer chromosomes for the two major autosomal chromosomes to estimate simultaneously the impact of different levels of inbreeding. In addition, they removed lethal genes from the analysis as these are expected to preclude the detection of a nonlinear accelerated rate of decline in fitness. With this approach they were also able to detect synergistic epistasis for induced mutations affecting egg-to-adult viability.

Here, we followed a classical full-sib mating approach and were also able to detect a non-linear decline for pupae productivity in two independent naturally segregating populations. A difference between our design and the previous successful ones mentioned above is that the latter implied crosses between lines, whereas we analysed the inbreeding depression occurred in a sample directly founded from a large segregating population, thus avoiding possible alterations in the frequencies of naturally segregating alleles and their linkage relationships. Our analysis also includes segregating lethal and highly deleterious mutations (at least in the initial generations) and refers to spontaneous rather than induced mutation, in contrast to the analysis of Sharp and Agrawal (2016). The non-linear accelerated decline in fitness was found both in log scale and raw scale, which implies that the results observed hold both under a multiplicative model and for an additive model of fitness variation. To avoid the problem of the loss of lines, we followed the recommendation of Lynch and Walsh (1998) of considering only the surviving lines at the end of the experiment. Not doing so would have implied that all LRT comparing linear and quadratic models were non-significant in all cases of Table 2 except for the case of Exp I in log scale for the genealogical inbreeding coefficient. The loss of non-linearity when all lines are considered (instead of just the surviving ones) is very apparent for Exp II, for which the decline in fitness became linear in log scale (Supplementary Fig. S8). The use of a proper outbred control in our experiments was also decisive to successfully detect a non-linear decline of fitness, as there was plenty of variation across inbred lines (Fig. 2), although this did not increase over generations of inbreeding as expected from neutral theory (i.e., as a function of 2*F*; Falconer and Mackay 1996), probably because of the loss of lines and genetic purging. Thus, it is possible that if these two design aspects would have been followed elsewhere, other inbreeding experiments which failed to detect synergistic epistasis could have been perhaps successful.

At the last generation (gen. 5 in Exp I with *F* = 0.633, and gen. 6 in Exp II with *F* = 0.703) the overall rate of inbreeding depression was a 1.20% and 1.32% decline in mean fitness per 1% increase in inbreeding, respectively, of the order of values found for the same trait in different populations of *Drosophila melanogaster* (García et al. 2012; Ávila et al. 2013) and also close to the average rate (1.32% ± 0.26) reported for many life-history traits (DeRose and Roff 1999).

Both experiments also showed a rather similar rate of loss of lines over generations, showing as well an accelerated rate upon inbreeding (Fig. 3) compatible with the rate of decline in fitness. The proportion of surviving lines at the last generation, with inbreeding coefficients of 0.6–0.7 was between 34 and 44%. This is in agreement with extinction rates obtained in other similar experiments. For example, Reed et al. (2003) carried out an experiment starting with 160 full-sib lines of *D. melanogaster* finding a proportion of surviving lines of 50% with *F* = 0.615 and of about 20% with *F* ≈ 0.8. Likewise, Wright et al. (2008) maintained an initial number of 107 full-sib lines of *D. simulans* for eight generations and the survival rate was around 30–40% at generations 6–7, i.e., with *F* ≈ 0.7–0.8.

In order to investigate the compatibility of different genetic models with the experimental results, we carried out computer simulations following the experimental design and assuming different epistatic models and a range of mutational parameters. For this we assumed mutation rates, effects and dominance of mutations within ranges compatible with empirical data from mutation-accumulation experiments (García-Dorado and Caballero 2000; Halligan and Keightley 2009; García-Dorado et al. 2004, Caballero 2017), as well as estimates obtained from genomic data on evolutionary divergence rates (Haag-Liautard et al. 2007; Charlesworth 2015). The assumption of a multiplicative model implied, as expected, a linear inbreeding depression for log fitness (Fig. 5). We first assumed a multi-locus epistatic model where homozygous effects interactions imply the squared of homozygous fitness values. This is compatible with the observation that fitness cost increases exponentially (approximately second order) with the number of accumulated mutations (Dickinson 2008). However, this model produced a slightly curvilinear decline in fitness for the full-sib lines, far from the observed results (Figs. S3 and S4).

We also considered a simpler model proposed by Charlesworth (1990) assuming quadratic effects on the number of heterozygous and homozygous genotypes. For one of the scenarios assumed (Table 1), the range of values for the parameter weighting the quadratic term (*ϕ*) covered the inferred estimates obtained by Charlesworth (1998) (*ϕ* = 0.0027) for viability in *D. melanogaster*. However, this model was not able to explain the non-linear decline in fitness observed for full-sib lines (Figs. S6 and S7). Our simulations, therefore, indicate that a quadratic fitness model does not provide, at least with fast inbreeding, results compatible with the observed non-linear decline for full-sib lines. This contrasts with simulations carried out by Salathé and Ebert (2003) assuming the same quadratic model as Charlesworth (1990), for which non-linear declines were shown for increasing inbreeding. However, Salathé and Ebert (2003) ignored purging selection in their simulations, and this is the most likely explanation for the difference between both sets of simulation results, as purging selection may erase the nonlinear effects of epistasis.

We found that a multi-locus interaction model, such that homozygous fitness values are raised to the power of the number of homozygous mutations (high-order homozygous fitness model) provided fitness declines similar to those observed (Fig. 5). When a high deleterious mutation rate was considered (*U* = 0.5), this model led to such a low fitness that full-sib lines could not be established. However, for a lower mutation rate of *U* = 0.05, the simulation results were close to the observations, particularly for those corresponding to intermediate selection coefficients *s* = 0.05–0.1 and average dominance coefficients *h* = 0.2–0.3, compatible with the empirical estimates obtained from mutation-accumulation experiments (García-Dorado and Caballero 2000; Halligan and Keightley 2009; García-Dorado et al. 2004; Caballero 2017). The simulation results close to the observed ones do not involve a very high order of epistatic effects. For example, for the results regarding *s* = 0.1; *h* = 0.2, homozygous epistatic effects were raised, on average, to a power of 2.0 at generation 3, a power of 3.0 at generation 5, and a power of 3.4 at generation 6.

Our results, in summary, suggest that synergistic epistasis of deleterious mutations not only occur among newly arisen spontaneous or induced mutations, but also among segregating alleles in natural populations, suggesting that synergistic epistasis may occur in natural populations. Most genetic variance for quantitative traits is expected to be additive, i.e., the relative magnitude of dominance and epistatic variances are generally much lower than that for additive variance (Hill et al. 2008). However, the lack of statistical epistasis is compatible with the pervasiveness of functional epistasis, that is, the molecular interaction between proteins or other genetic elements (de Visser and Hoekstra 1998; Phillips 2008; de Visser et al. 2011; Mackay 2013; Sohail et al. 2017). Epistasis may take a role in a number of relevant issues in evolutionary biology, such as the evolution of sex and recombination, but also in problems regarding the constitution of quantitative traits, such as the observed gap between the heritabilities explained by variants detected in Genome-Wide Association Studies (GWAS) and the familial estimates of heritability for human traits (Manolio et al. 2009; Hemani et al. 2013). In a large meta-analysis of the heritability of human traits, Polderman et al. (2015) found that genetic variation for about 30% of the traits cannot be exclusively explained by an additive model. Thus, at least for some traits, estimates of familial heritability obtained from twin studies can be inflated by epistatic and environmental components of variance (Zuk et al. 2012), perhaps contributing to the gap between narrow-sense GWAS heritabilities and familial broad-sense heritabilities (López-Cortegano and Caballero 2019).

Although we have interpreted our observed results as a support for synergistic epistasis, other alternative explanations are, however, possible. First, in our simulations we assumed that all inbreeding depression is due to the homozygous effects of deleterious mutations. It is possible that balancing selection is partly responsible for the observed fitness decline (Charlesworth 2015), and the impact of epistasis on inbreeding depression may be different for this model and the partial dominance model. For example, inbreeding purge can only occur under the partial dominance hypothesis of inbreeding depression (Benesh et al. 2014). Second, it has been suggested that mobilisation of transposable elements may be a possible cause of the accelerated decline in viability due to insertional mutations, rather than synergistic epistasis, in Mukai´s (1969) mutation-accumulation experiment (Keightley 1996). Inbreeding is one of the several factors known to produce genomic instability (Yurchenko et al. 2011; García Guerreiro 2012), so it is possible that an increased mobilisation of transposable elements occurs also in highly inbred lines, mimicking the nonlinear decline in fitness observed. Finally, inbreeding has been shown to produce substantial changes in gene expression (Kristensen et al. 2005; Ayroles et al. 2009; Paige 2010; García et al. 2012), which could modulate the rate of inbreeding depression. In fact, it has been shown that some changes of gene expression triggered by inbreeding may have a protective role against the negative effects of inbreeding, in the sense of restricting the amount of inbreeding depression for fitness (García et al. 2012, 2013a, 2013b). Thus, it is possible that accelerated declines for fitness under inbreeding are a consequence of changes in expression across the genome. In summary, our results are compatible with the existence of synergistic epistasis between deleterious recessive alleles, but other alternative explanations cannot be discarded and further studies are necessary to evaluate them.

### Data archiving

Data have been submitted to https://github.com/armando-caballero/Dominguez-et-al-Heredity.

## Supplementary information

Supplemental Material
