# Supplementary material for: Accelerated inbreeding depression suggests synergistic epistasis for deleterious mutations in Drosophila melanogaster
Source: Heredity (Edinb). 2019 Sep 2;123(6):709–22. doi: 10.1038/s41437-019-0263-6 (PMC6834575; doi:10.1038/s41437-019-0263-6)

**SUPPLEMENTARY TABLES AND FIGURES**

**Table S1**. Intercept, linear and quadratic coefficients of the quadratic regressions fitted to the observed fitness decline in Experiments I and II using raw or log scale data and assuming both the genealogical inbreeding coefficients (*F*; Figures 4A and 4B) or purged inbreeding coefficients (*g*; Supplementary Figures S2A and S2B).

|  | **Genealogical inbreeding (*F*)** | |  | **Purged inbreeding (*g*)** | |
| --- | --- | --- | --- | --- | --- |
| **Experiment** | **I** | **II** |  | **I** | **II** |
| **RAW SCALE** |  |  |  |  |  |
| **Intercept** | 1.01 | 0.99 |  | 1.01 | 0.98 |
| **Coeff. on *F* or *g*** | 0.10 | –0.35 |  | 0.25 | 0.00 |
| **Coeff. on *F*^2^ or *g*^2^** | –1.42 | –0.67 |  | –2.21 | –1.76 |
| **LOG SCALE** |  |  |  |  |  |
| **Intercept** | –0.01 | –0.03 |  | –0.02 | –0.04 |
| **Coeff. on *F* or *g*** | 0.66 | 0.04 |  | 1.01 | 0.62 |
| **Coeff. on *F*^2^ or *g*^2^** | –2.79 | –1.78 |  | –4.30 | –3.65 |

**Table S2**. Probabilities for a normality test of the data. The bold figure indicates the only test showing a significant departure from normality.

| **Experiment** | **I** | **II** | **I + II** |
| --- | --- | --- | --- |
| **RAW SCALE** |  |  |  |
| **P. normal (linear)** | 0.44 | 0.09 | **0.03** |
| **P. normal (quad.)** | 0.51 | 0.10 | 0.21 |
| **LOG SCALE** |  |  |  |
| **P. normal (linear)** | 0.37 | 0.69 | 0.21 |
| **P. normal (quad.)** | 0.51 | 0.15 | 0.32 |

**Figure S1.** Relationship between the expected purged inbreeding coefficient (*g*), obtained assuming a purge coefficient of *d* = 0.1, and the expected genealogical inbreeding coefficient (*F*) in the experiment. The dotted line represents identity between both coefficients.


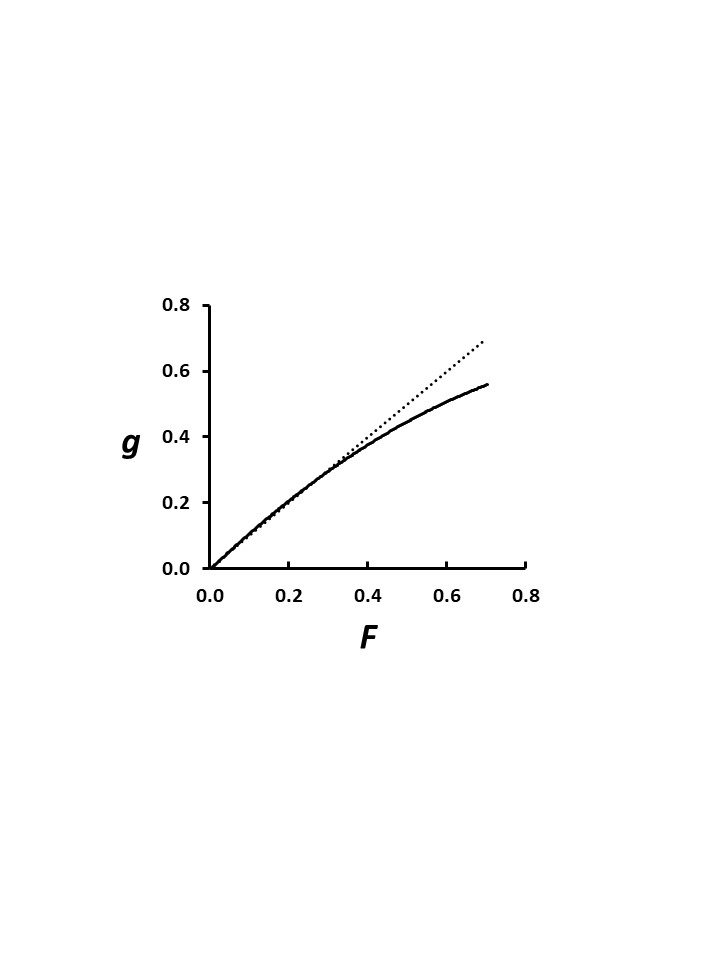


**Figure S2.** Inbreeding depression for pupae productivity for Experiments I and II, estimated as the ratio between the average productivity of inbred lines (*W_I_*) and the outbred control (*W_O_*) in each generation (panel A) or the logarithm of the ratio (panel B), against the expected purged inbreeding coefficient (*g*). The data fitted better to a quadratic line (shown) than to a linear one.

**
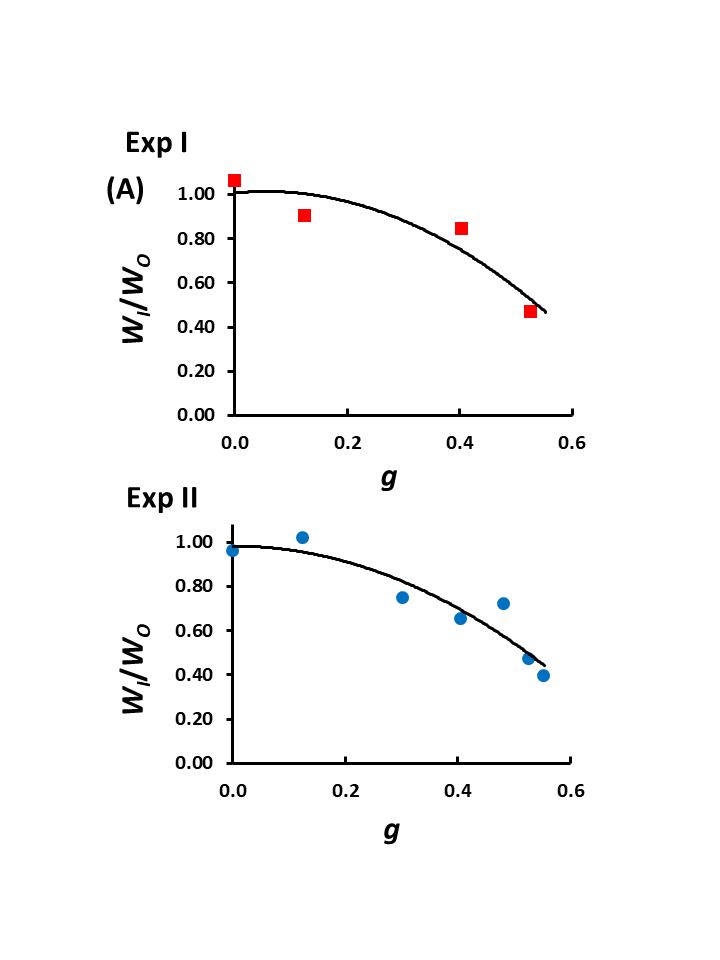

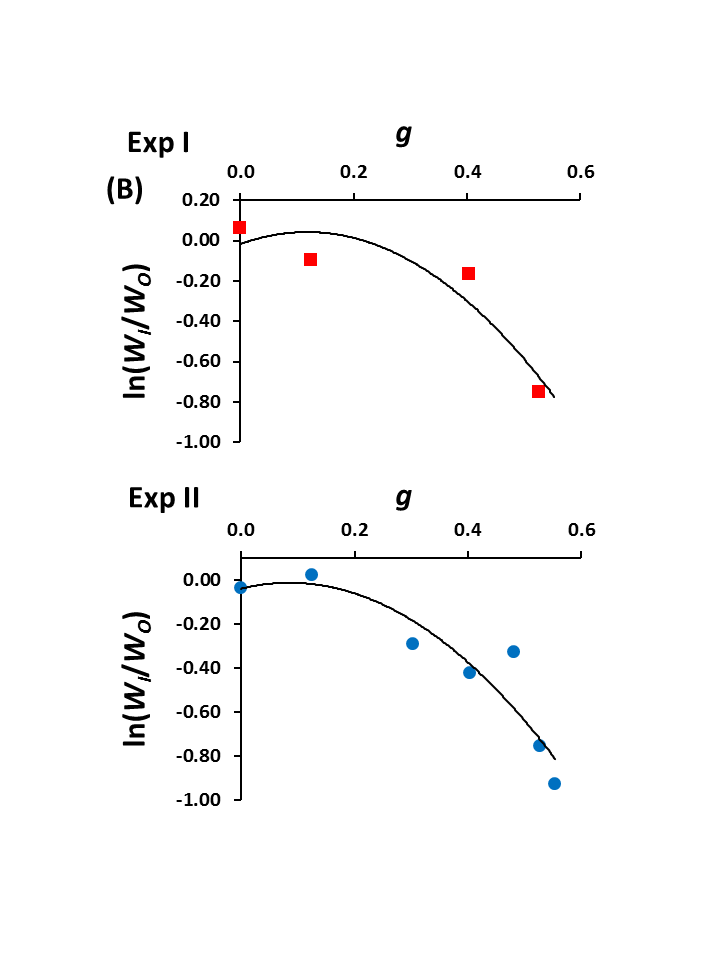
**

**Figure S3.** Comparison between simulation results (lines), assuming the quadratic homozygous fitness model, and experimental data (dots) including Experiments I and II. Each panel shows the decline in log relative fitness, ln(*W_I_*), for increasing values of the expected genealogical inbreeding coefficient (*F*) in the full-sib lines. Averaged simulation results assuming a multiplicative (non-epistatic) model are shown as dotted thin lines, whereas averaged results assuming an epistatic model of variation are shown as broken thick lines. The epistatic model assumes that homozygous fitnesses of epistatic loci are squared. Deleterious mutations are assumed to appear with haploid rate *U* = 0.1, variable effects obtained from a gamma distribution with shape parameter *β* = 2, mean homozygous effect *s* and variable dominance coefficients with mean *h*.


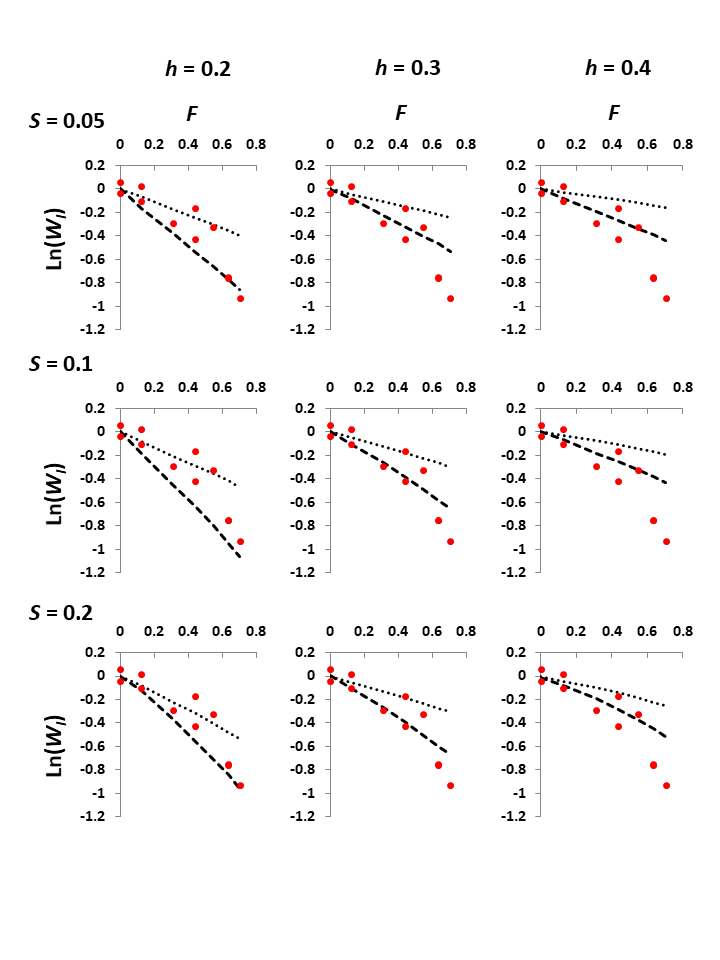


**Figure S4.** Comparison between simulation results (lines), assuming the quadratic homozygous fitness model, and experimental data (dots) including Experiments I and II. Each panel shows the decline in log relative fitness, ln(*W_I_*), for increasing values of the expected genealogical inbreeding coefficient (*F*) in the full-sib lines. Averaged simulation results assuming a multiplicative (non-epistatic) model are shown as dotted thin lines, whereas averaged results assuming an epistatic model of variation are shown as broken thick lines. The epistatic model assumes that homozygous fitnesses of epistatic loci are squared. Deleterious mutations are assumed to appear with haploid rate *U* = 0.5, variable effects obtained from a gamma distribution with shape parameter *β* = 0.2, mean homozygous effect *s* and variable dominance coefficients with mean *h*.


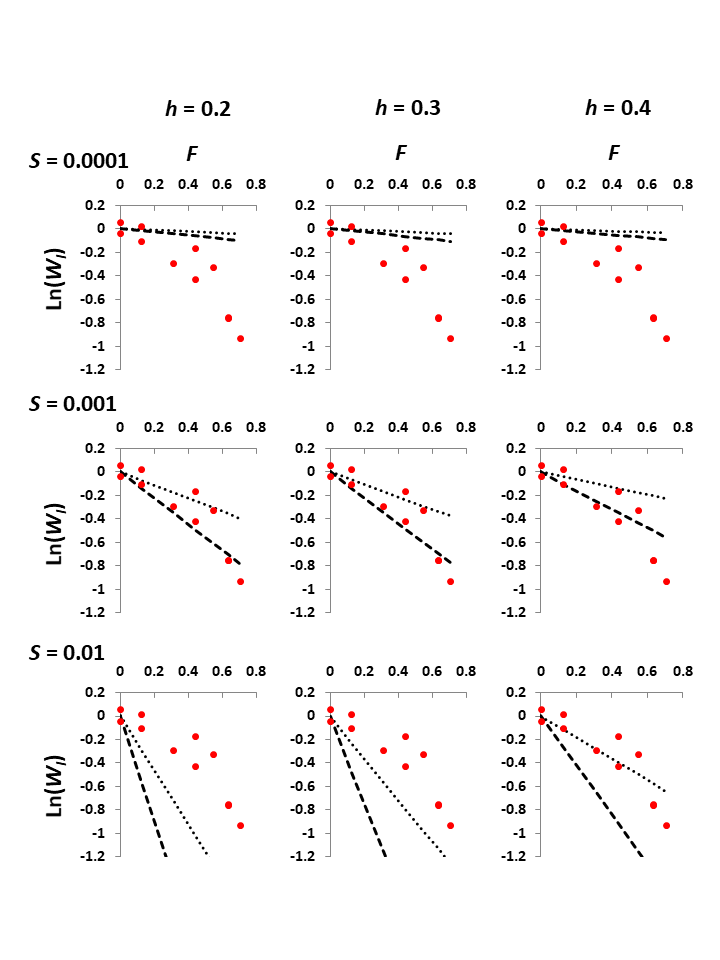


**Figure S5.** Ratio between the average frequency of homozygous deleterious mutations and the corresponding frequency assuming there is no purging selection in simulated full-sib lines assuming the high order homozygous fitness epistatic model. The mutational parameters assumed are: haploid mutation rate *U* = 0.05, variable effects obtained from a gamma distribution with shape parameter *β* = 2, mean homozygous effect *s* = 0.1 and variable dominance coefficients with mean *h* = 0.2.


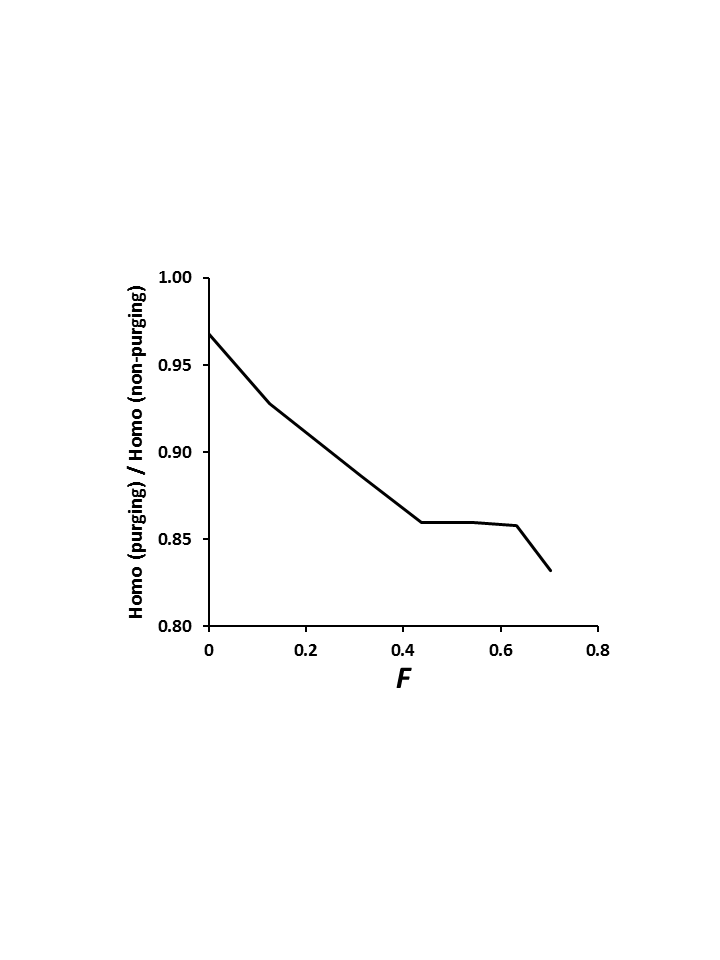


**Figure S6.** Comparison between simulation results (lines) and experimental data (dots) including Experiments I and II. Each panel shows the decline in log relative fitness, ln(*W_I_*), for increasing values of the expected genealogical inbreeding coefficient (*F*) in the full-sib lines. Averaged simulation results assuming a model in which the fitness of individual *i* is

*W_i_* = exp[−*α*(*n*_1_ + *hn*_2_) – ½*ϕ*(*n*_1_ + *hn*_2_)^2^, where *n*_1_ and *n*_2_ are the number of heterozygous and homozygous loci in the individual, respectively, *h* is the constant coefficient of dominance assumed (0.2), and *α* and *ϕ* are positive coefficients which determine the impact of mutations on fitness (linear and quadratic). Deleterious mutations are assumed to appear with haploid rate *U* = 0.05.


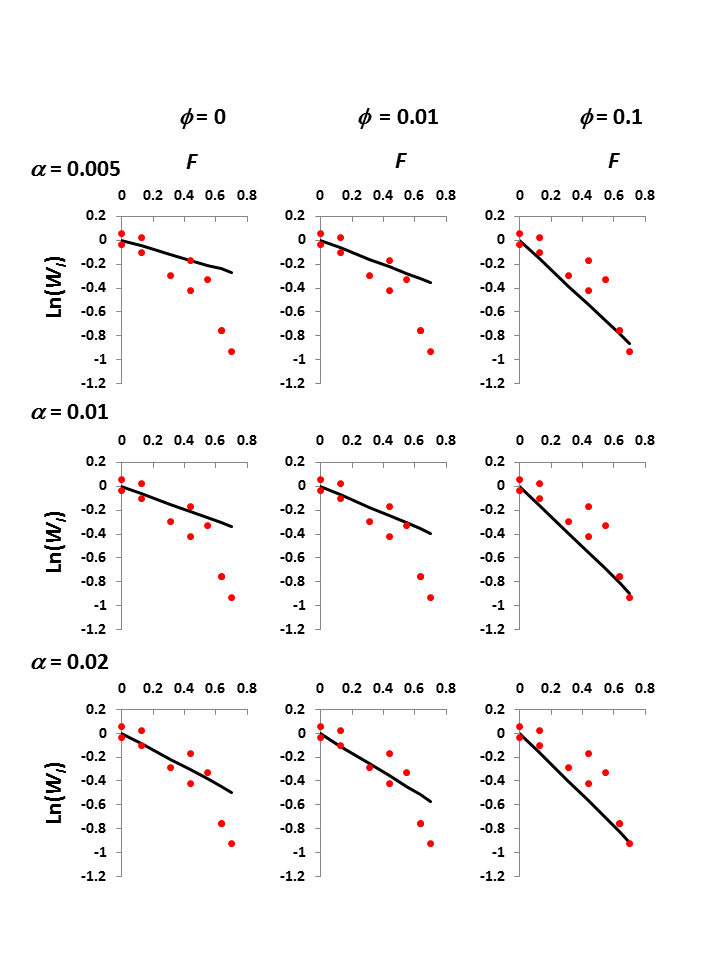


**Figure S7.** Comparison between simulation results (lines) and experimental data (dots) including Experiments I and II. Each panel shows the decline in log relative fitness, ln(*W_I_*), for increasing values of the expected genealogical inbreeding coefficient (*F*) in the full-sib lines. Averaged simulation results assuming a model in which the fitness of individual *i* is

*W_i_* = exp[−*α*(*n*_1_ + *hn*_2_) – ½*ϕ*(*n*_1_ + *hn*_2_)^2^, where *n*_1_ and *n*_2_ are the number of heterozygous and homozygous loci in the individual, respectively, *h* is the constant coefficient of dominance assumed (0.2), and *α* and *ϕ* are positive coefficients which determine the impact of mutations on fitness (linear and quadratic). Deleterious mutations are assumed to appear with haploid rate *U* = 0.5.


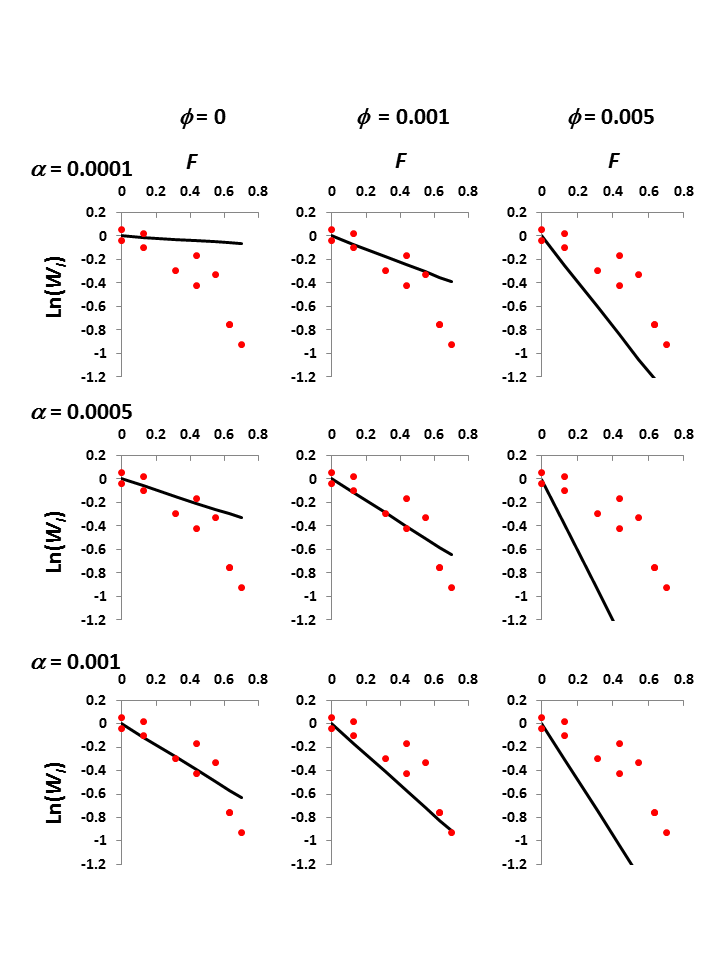


**Figure S8.** Inbreeding depression for pupae productivity for Experiments I and II, estimated as the ratio between the average productivity of inbred lines (*W_I_*) and the outbred control (*W_O_*) in each generation (panel A) or the logarithm of the ratio (panel B), against the expected genealogical inbreeding coefficient (*F*). The analysis includes all full-sib lines instead of only the surviving lines (cf. Figure 4 of main text).


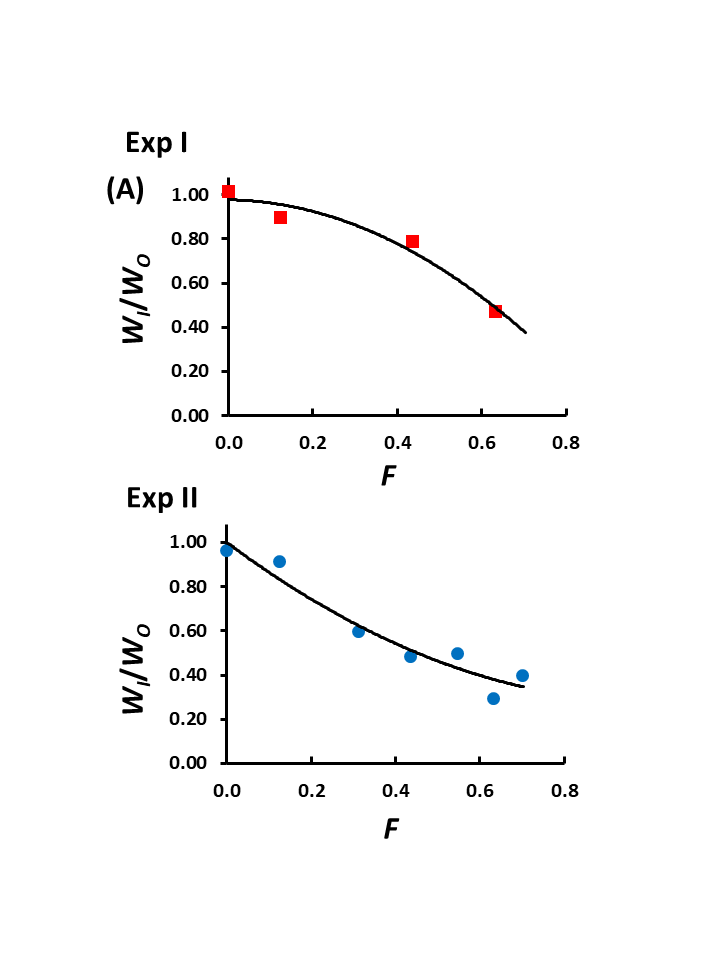

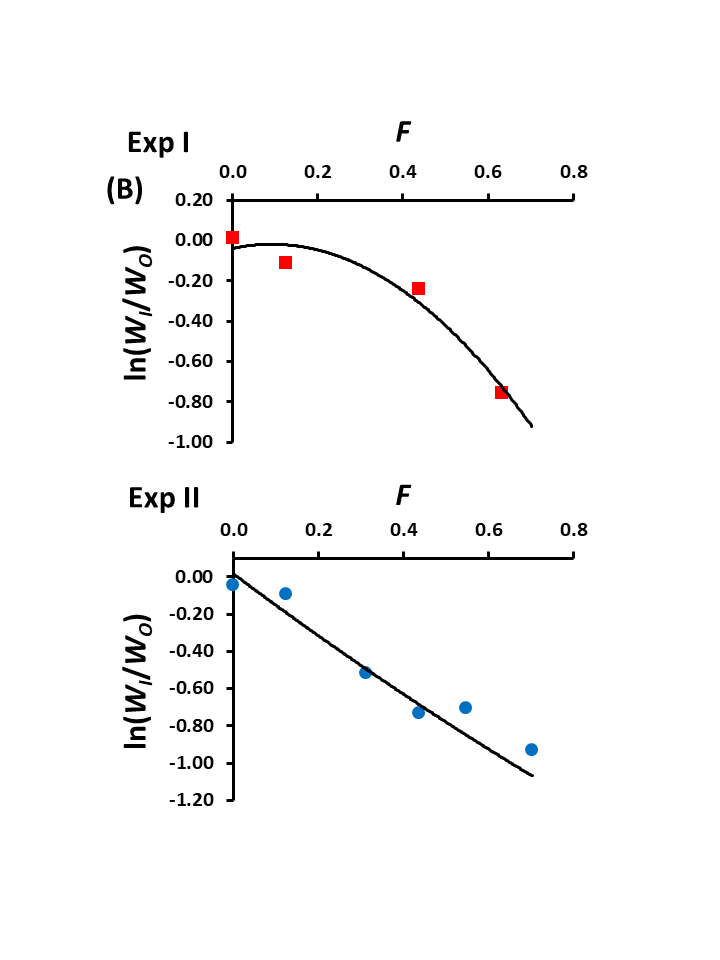

Supplement: Supplementary file 1 — Supplemental Material [file 41437_2019_263_MOESM1_ESM.docx]
